# Synthesis of Tellurium Nanoparticles Using *Moringa oleifera* Extract, and Their Antibacterial and Antibiofilm Effects against Bacterial Pathogens

**DOI:** 10.3390/microorganisms12091847

**Published:** 2024-09-06

**Authors:** Bo Ao, Honglin Jiang, Xuan Cai, Decheng Liu, Junming Tu, Xiaoshan Shi, Yanxiang Wang, Fei He, Jing Lv, Jingjing Li, Yuanliang Hu, Xian Xia, Jianjun Hou

**Affiliations:** 1 Hubei Key Laboratory of Edible Wild Plants Conservation & Utilization, Hubei Engineering Research Center of Characteristic Wild Vegetable Breeding and Comprehensive Utilization Technology, Hubei Normal University, Huangshi 435002, China; aobo99123@163.com (B.A.); 15667172820@163.com (D.L.); junming_tu@hbnu.edu.cn (J.T.); shixs@hbnu.edu.cn (X.S.); wangyx@hbnu.edu.cn (Y.W.); pandali1980@hotmail.com (J.L.); ylhu@hbnu.edu.cn (Y.H.); 2 Hubei Key Laboratory of Natural Medicinal Chemistry and Resource Evaluation, School of Pharmacy, Tongji Medical College, Huazhong University of Science and Technology, Wuhan 430030, China; 3 Hubei Provincial Center for Disease Control and Prevention, Wuhan 430079, China; jhl13098825387@163.com (H.J.); 13036167687@163.com (F.H.); lvjing979899@163.com (J.L.); 4 Wuhan University, Wuhan 430060, China; rm001319@whu.edu.cn

**Keywords:** *Moringa oleifera*, Bio-TeNPs, nanomaterials, antibacterial, antibiofilm

## Abstract

Today, pathogenic microorganisms are increasingly developing resistance to conventional drugs, necessitating the exploration of alternative strategies. In addressing this challenge, nano-based antibacterial agents offer a promising avenue of research. In the present study, we used an extract of *Moringa oleifera*, a widely recognized edible and medicinal plant, to synthesize biogenetic tellurium nanoparticles (Bio-TeNPs). Transmission electron microscopy, scanning electron microscopy, and dynamic light scattering analyses revealed that the obtained Bio-TeNPs had diameters between 20 and 50 nm, and zeta potential values of 23.7 ± 3.3 mV. Fourier-transform infrared spectroscopy and X-ray photoelectron spectroscopy revealed that the Bio-TeNPs consisted primarily of Te(0), along with some organic constituents. Remarkably, these Bio-TeNPs exhibited potent antibacterial activity against a spectrum of pathogens, including *Escherichia coli*, *Klebsiella pneumoniae*, *Shigella dysenteriae*, *Salmonella typhimurium*, *Streptococcus pneumoniae*, and *Streptococcus agalactiae*. In addition, findings from growth curve experiments, live/dead cell staining, and scanning electron microscopy observations of cell morphology demonstrated that Bio-TeNPs at a concentration of 0.07 mg/mL effectively disrupted *E*. *coli* and *K*. *pneumoniae* cells, leading to cell rupture or shrinkage. The biofilm inhibition rates of 0.7 mg/mL Bio-TeNPs against *E*. *coli* and *K*. *pneumoniae* reached 92% and 90%, respectively. In addition, 7 mg/mL Bio-TeNPs effectively eradicated *E*. *coli* from the surfaces of glass slides, with a 100% clearance rate. These outcomes underscore the exceptional antibacterial efficacy of Bio-TeNPs and highlight their potential as promising nanomaterials for combating bacterial infections.

## 1. Introduction

Bacterial infections currently stand as the second-leading cause of death worldwide, significantly impacting both human health and economic development [1]. Unfortunately, the irrational use of antibiotics and the natural selection of bacteria have exacerbated the problem of drug-resistant strains [2]. Consequently, there is an urgent need to explore novel therapeutic avenues to combat bacterial infections. Fortunately, nanotechnology has been rapidly advancing in the realm of biomedicine, offering a promising approach to address both bacterial infections and drug resistance [3]. Bionanomaterials, which are distinguished by their enhanced bioactivity and biocompatibility, compared with their physically and chemically synthesized counterparts, stand out as particularly promising candidates in this regard. Various antibacterial nanomaterials, including AgNPs [4], AuNPs [5], CuONPs [6], and ZnONPs [7], have demonstrated high levels of efficacy in combating bacterial infections. Compared with conventional methods which are reliant on hazardous chemicals and involve the production of toxic by-products [8], biological synthesis methods offer a safer and more environmentally friendly alternative [9]. The production of bionanomaterials is characterized by mild conditions, safe processes, and overall eco-friendliness [10]. Bionanomaterials are also easy to synthesize [11]. In general, microorganisms and plant extracts are used to synthesize bionanomaterials [12]. Compared with microorganisms, edible and medicinal plants offer a richer and safer array of active metabolites. Previous research has indicated that natural active products in plant extracts could be more effectively combined with nanomaterials so that they gain additional biological functions [13].

*Moringa oleifera* is an edible medicinal plant native to India [14]; its extracts contain various bioactive properties that are now being effectively exploited [15]. *M. oleifera* is currently used to produce functional nanomaterials, such as AgNPs [16,17], MgONPs [18,19], SeNPs [20], and ZnONPs [21], which exhibit antibacterial, antioxidant, and anticancer properties. Tellurium (Te) is a semimetallic element in the chalcogen family, and commonly exists in nature in oxidized states of −II, 0, +IV, and +VI, with Te(IV) and Te(VI) as its main forms [22]. Te compounds were first used as antimicrobial agents as early as 1932. These compounds exert their antimicrobial properties mainly in the form of tellurite ions [23]. Additionally, tellurium nanomaterials have also been used for applications in biomedicine on account of their antimicrobial, antioxidant, antifungal, and anticancer properties [24]. In recent years, biosynthesis of the nanoforms of Te has attracted the attention of many researchers. For instance, *Pseudoalteromonas shioyasakiensis* [25] and *Mortierella* sp. AB1 [26] were used to synthesize biogenetic Te nanoparticles (Bio-TeNPs), which inhibited various pathogenic bacteria. Similarly, *Streptomyces cyaneus* [27] and *Gayadomonas* sp. TNPM15 were used to produce Bio-TeNPs which repressed certain *Aspergillus* pathogens and phytopathogenic fungi [28]. Studies such as these have demonstrated the excellent antibacterial properties of Bio-TeNPs. In addition, extracts of aloe vera [29], pepper [30], and citrus fruit [31] have been used to synthesize antibacterial and anticancer Bio-TeNPs. However, the use of *M. oleifera* extract to synthesize Bio-TeNPs has not yet been reported.

The thin branches and discarded shredded leaves of *M. oleifera* have lower economic value, compared with the seeds and flowers. The use of such by-products for nanomaterial production could increase their value as plant material, while at the same time reducing the cost of synthesizing Bio-TeNPs. In the present study, the extract was taken from thin branches and discarded shredded leaves of *M. oleifera*; this was used as a stabilizing, capping, and reducing agent to synthesize Bio-TeNPs. These Bio-TeNPs were then characterized by visible light spectrum analysis, transmission electronic microscopy (TEM), scanning electron microscopy (SEM), dynamic light scattering (DLS), zeta potential analysis, energy-dispersive spectroscopy (EDS), X-ray diffraction (XRD), Fourier-transform infrared spectroscopy (FTIR), and X-ray photoelectron spectroscopy (XPS). In addition, the antibacterial activity of Bio-TeNPs was tested in a series of experiments and then evaluated.

## 2. Materials and Methods

### 2.1. Materials

*M. oleifera* samples were obtained from Hubei Mingshan Jingnan Moringa Technology Co., Ltd. (Huangshi, China). Potassium tellurite (K_2_TeO_3_) was purchased from Shanghai Aladdin Biochemical Technology (Shanghai, China) Co., Ltd. Chemical element tellurium [Chem-Te(0)] (CAS No. 13494-80-9) was obtained from Shanghai Yien Chemical Technology (Shanghai, China) Co., Ltd. The strains *Escherichia coli* ATCC 25922, *Klebsiella pneumoniae* ATCC 700603, *Shigella dysenteriae* CMCC 51252, *Salmonella typhimurium* ATCC 14028, *Streptococcus pneumoniae* ATCC 49619, *Streptococcus agalactiae* E442, and *Staphylococcus aureus* ATCC 25923 were obtained from the Strain Preservation Center of Hubei Centers for Disease Control and Prevention (Wuhan, China). Bacteria were cultured on MHA (Mueller–Hinton Agar) medium at 37 °C.

### 2.2. Biosynthesis of Bio-TeNPs

Fresh specimens of discarded thin branches and leaves underwent a process of washing, drying, and storage at room temperature. A quantity of 100 g of these thin branches and leaves was then combined with 1000 mL of distilled water (ddH_2_O) and brought to a boil for 5–10 min. Next, the mixture was filtered, and the resulting liquid was stored at 4 °C for 24 h before use. Subsequently, a 50 mL portion of the extract was blended with 0.1 g of K_2_TeO_3_ in a 100 mL glass beaker at 28 °C for 4 h, using a magnetic stirrer. The resulting suspension was centrifuged at 12,000 rpm for 5 min and washed three times with ddH_2_O. Subsequently, the washed tellurite nanoparticles were resuspended in 10 mL of ddH_2_O. The resulting material was dried, and the concentrations of Bio-TeNPs were then determined.

### 2.3. Characterization of Bio-TeNPs

To gain insights into the morphology and composition of Bio-TeNPs, the Bio-TeNPs produced as described in Section 2.2 were subjected to further analysis using a previously established protocol [26]. The Bio-TeNPs solution underwent examination using a plate reader within a wavelength range of 300–800 nm (SpectraMax i3x, Molecular Devices, CA, USA). Subsequently, the Bio-TeNPs solution was sonicated, applied onto a copper mesh, dried, and processed for observation and EDS analysis under transmission electron microscopy (Tecnai G2 F30, FEI, Hillsboro, OR, USA). Additionally, the Bio-TeNPs solution was sonicated, diluted, and introduced into an electrophoresis system with instrumental parameters set to 25 °C for 10 s to determine its zeta potential (Zetasizer Nano ZS90, Malvern, UK). Simultaneously, a portion of the same liquid was transferred to a cuvette, with instrument parameters set to 25 °C for 10 s at an angle of 173° to measure dynamic light scattering (Zetasizer Nano ZS90, Malvern, UK). Following these measurements, the Bio-TeNPs were subjected to drying using an SJIA-10N vacuum freeze dryer (Ningbo YinZhou Sjia Lab Equipment Co., Ltd., Yinzhou, China). The dried Bio-TeNPs were then affixed on a tray coated with gold and observed using a scanning electron microscope (SU8010, Hitachi, Tokyo, Japan). Dried Bio-TeNPs were also tested using X-ray diffraction (Bruker D8 Advance, GER, Billerica, MA, USA) with a scan time of 10 min and a scan rate of 10°/min. Further analysis involved pressing the dried Bio-TeNPs, setting the spectral range to 400–4000 cm^−1^, and ensuring a resolution accuracy of 0.4 cm^−1^ using infrared spectroscopy (Nicolet IS10, Thermo Fisher, Waltham, MA, USA). Dried Bio-TeNPs were also pressed and placed in an apparatus for X-ray photoelectron spectroscopy (ESCALAB 250XI, Thermo Fisher Scientific, Waltham, MA, USA) with a pass energy of 30 eV (0.10 eV) and 120 eV (1.00 eV) at a spot size of 500 µm. Under these conditions, upon excitation by X-rays, photoelectron data were collected to form an energy spectrum.

### 2.4. Antibacterial Activity Tests

To assess antibacterial activity, inhibition zone tests were performed. First, the chosen bacteria were cultured on MHA medium overnight at 37 °C. Following incubation, bacterial colonies from the MHA plates were harvested, washed, and suspended in 0.85% (*w*/*v*) saline solution. These suspensions were then diluted to a turbidity of 0.5 McFarland standard (~1 × 10^8^ cfu/mL) using a turbidity meter (Vitek 2 DensiCHEK) (bioMérieux, Lyon, France). The diluted bacterial suspensions were evenly spread onto MHA plates (50 µL bacterial suspension per plate). Subsequently, sterilized Oxford cups were placed on each plate, and varying concentrations of Bio-TeNPs (0.07, 0.7, and 7 mg/mL) were added to the cups (100 µL each). As a control, an extract of M. oleifera (7 mg/mL) and Chem-Te (0) (7 mg/mL) was utilized. The plates were then incubated at 37 °C for 15 h, allowing for the inhibition of bacterial growth. Following incubation, the diameters of the inhibition zones surrounding the Oxford cups were measured using vernier calipers; these diameters were recorded as indicators of antibacterial efficacy.

### 2.5. Growth Curve Experiments

To further evaluate the antibacterial efficacy of Bio-TeNPs, a growth curve analysis was conducted. Bacteria, cultured overnight on MHA plates as outlined in Section 2.4, were harvested and suspended in MHB media. A fresh suspension of *E*. *coli* and *K*. *pneumoniae* with a turbidity of 0.6 McFarland units was prepared using a turbidity meter (Vitek 2 DensiCHEK). Next, 50 µL of the suspension was added to 5 mL of MHB medium. The growth of *E*. *coli* and *K*. *pneumoniae* in MHB media was then assessed in the presence of Bio-TeNPs, Chem-Te(0), and *M*. *oleifera* extract at concentrations of 0.07, 0.035, and 0.007 mg/mL. Bacteria cultured in an MHB medium without any samples served as the control group. Next, 200 µL samples were collected every 2 h, and their optical density was measured at 600 nm using a microplate reader (SpectraMax i3x, Molecular Devices, CA, USA).

### 2.6. Live/Dead Cell Staining

To investigate the bactericidal effect of Bio-TeNPs on *E. coli* and *K. pneumoniae*, a live/dead cell staining experiment was conducted. Bacteria, cultured overnight on MHA plates as detailed in Section 2.4, were harvested and resuspended in MHB media to achieve a turbidity of 0.5 McFarland standard (~1 × 10^8^ cfu/mL). These bacterial suspensions were then cultured with Bio-TeNPs at concentrations of either 0 or 0.07 mg/mL in a shaker at 150 rpm, incubated at 37 °C for 4 h. Following incubation, the cells were collected and washed with 0.85% (*w*/*v*) saline solution. A LIVE/DEAD Bacterial Staining Kit (BBcellProbe^®^ N01/PI, BestBio, Shanghai, China) was utilized to stain the live and dead cells according to the manufacturer’s instructions [32]. In brief, the bacteria were resuspended in PI and NO1 dye working solution, followed by incubation away from light for 15 min. Subsequently, the bacteria were washed and resuspended in 0.85% saline solution. Finally, a 5–10 μL quantity of bacterial suspension was taken and added to a slide for sample observation. The stained bacteria were excited at 488 nm, and live and dead cells were observed using laser scanning confocal microscopy (Nikon Eclipse Ti, Tokyo, Japan).

### 2.7. Morphological Observation of Microorganisms

Bacteria (~1 × 10^8^ cfu/mL) were cultured in the MHB media at 0.07 mg/mL of Bio-TeNPs, with ddH_2_0, Chem-Te(0) and *M. oleifera* extract as control groups. The cultures were incubated at 150 rpm at 37 °C for 4 h. After incubation, 2 mL amounts of cultures were collected and processed according to previously described methods [20]. Briefly, the collected samples were fixed overnight at 4 °C in 2.5% glutaraldehyde, washed three times with 1.5 mL of physiological saline solution, and then dehydrated with a gradient of anhydrous ethanol (30%, 50%, 70%, 80%, 90%, 100%). Next, the obtained samples were freeze-dried for 12–24 h. The bacteria were then immobilized on trays and treated with gold spray. The treated *E*. *coli* and *K*. *pneumoniae* were observed via SEM (S-4800, Hitachi, Tokyo, Japan).

### 2.8. Biofilm Formation Inhibition Assay

Crystalline violet staining and fluorescein isothiocyanate (FITC) staining were used to determine the effect of compounds on *E*. *coli* and *K*. *pneumoniae* biofilm formation and attachment. According to the method described in Section 2.4, fresh *E*. *coli* and *K*. *pneumoniae* suspensions of 0.6 M were prepared using a turbidity meter (Vitek 2 DensiCHEK). A 20 µL amount of bacterial solution and a 180 µL amount of MHB medium containing different concentrations of Bio-TeNPs (0.007, 0.035, 0.07, and 0.7 mg/mL), extract of *M. oleifera* (0.07 and 0.7 mg/mL), and Chem-Te (0) (0.007, 0.035, 0.07, and 0.7 mg/mL) were added to the 96-well plate, mixed uniformly and incubated statically at 37 °C for 24. The bacterial biofilm formation was then washed using PBS, fixed in methanol, and stained with crystal violet and solubilized 33% (*v*/*v*) acetic acid solution. In addition, a portion of cultured bacteria was collected and washed with PBS to remove planktonic bacteria. Subsequently, bacteria were stained with FITC for 30 min and washed with PBS to remove unbound dye. Finally, stained bacteria were observed under an inverted fluorescence microscope (Nikon Eclipse Ti 2, Tokyo, Japan).

### 2.9. Clearance Rate of Bio-TeNPs against E. coli on the Surfaces of Glass Slides

Bacteria, cultured overnight on MHA plates as detailed in Section 2.4, were harvested and resuspended in MHB media. The *E*. *coli* suspension (30 µL, ~2 × 10^8^ cfu/mL) was evenly spread onto a glass slide measuring 2 cm × 2 cm and allowed to air-dry for 15 min to fix the bacteria. Subsequently, the area covered with the bacterial specimen was treated with 100 µL of Bio-TeNPs (7 and 0.7 mg/mL), Chem-Te(0) (7 and 0.7 mg/mL), *M*. *oleifera* extract (7 mg/mL), and 0.85% (*w*/*v*) normal saline; this was dispensed using pipettes, the tips of which were used to achieve an even spread. After 20 min, cells were recovered from the glass slides by scraping with cotton swabs, which were then immersed in 1 mL of 0.85% (*w*/*v*) normal saline. Finally, 50 µL of the aforementioned treated normal saline stock solutions and dilutions (10^−1^, 10^−2^, and 10^−3^) were spread onto MHA plates. The number of colonies was enumerated to determine the bacterial survival rate.

### 2.10. Cytotoxicity Evaluation of Bio-TeNPs

In order to evaluate the cytotoxicity of Bio-TeNPs, vero-E6 cells were used as a cell model, determined by the Cell Counting Kit-8 (CCK-8) (APExBIO, Houston, TX, USA). First, Vero-E6 cells were inoculated in a 96-well culture plate (1.0 × 10^4^ cells/100 μL per well) for 24 h at 37 °C, 5% CO_2_. Then, Bio-TeNPs were added to 96-well plates with different final concentrations (7, 0.7, and 0.07 mg/mL). Chem-Te(0) (7 mg/mL) and Dulbecco’s modified Eagle’s medium (DMEM) (7 mg/mL) were used as control groups. After 24 h, the culture medium was removed and washed twice with PBS. Finally, to each well of the plate was added 100 μL fresh DMEM and 10 μL CCK-8; incubation then continued for 3 h at 37 °C. The absorbance at 450 nm was determined using a multi-plate reader (SpectraMax i3x, Molecular Devices, San Jose, CA, USA).

## 3. Results

### 3.1. Characterization of Bio-TeNPs

**Figure 1:**
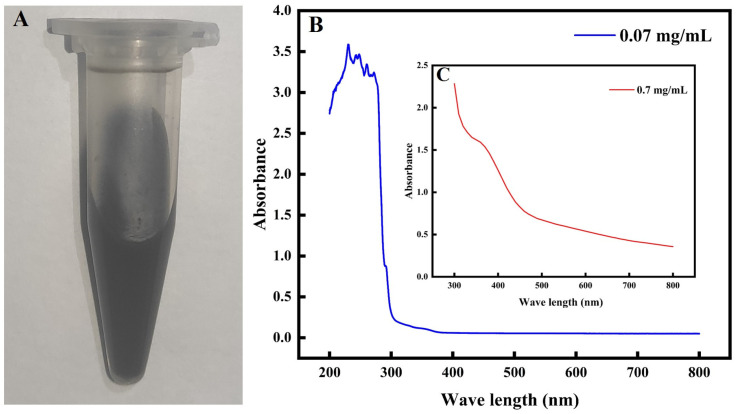
Appearance (**A**) and visible absorbance spectrum (**B**,**C**) of Bio-TeNPs (0.07 and 0.7 mg/mL).

**Figure 2:**
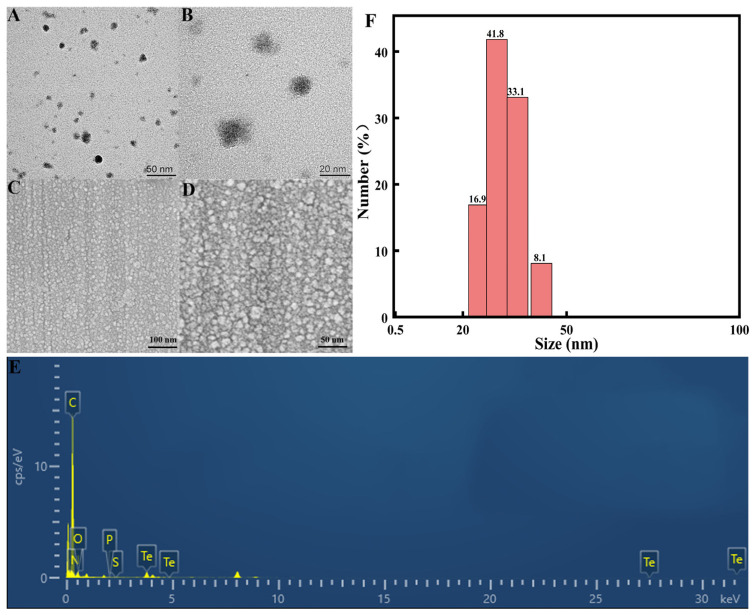
The morphology and size of Bio-TeNPs. Image of Bio-TeNPs with a diameter of 20 to 50 nm obtained using TEM (**A**,**B**), SEM (**C**,**D**), EDS (**E**), and DLS (**F**).

The Bio-TeNPs were typically black in color (Figure 1A). The UV-visible spectrum of the Bio-TeNPs was analyzed using a microplate reader. A number of peaks noticeable at approximately 230 nm, 250 nm, 260 nm, and a slight peak at 350 nm were observed at 0.07 mg/mL of Bio-TeNPs (Figure 1B). At a concentration of 0.7 mg/mL of Bio-TeNPs, a distinct peak at 350 nm was observed (Figure 1C). To identify the morphology and size of the Bio-TeNPs in this study, TEM, SEM, DLS, EDS, and XRD were performed. The results indicated that the Bio-TeNPs had diameters of 20 to 50 nm and irregular spherical shapes (Figure 2A–D). Some of the Bio-TeNPs in the SEM were slightly larger, probably because some of the nanoparticles agglomerated together during the freeze-drying process. The zeta potential of the Bio-TeNPs was found to be 23.7 ± 3.3 mV. The EDX result indicated that the composition of Bio-TeNPs was rich in tellurium. In addition, the XRD spectrum showed Te crystal peaks at (100), (101), (102), and (110) (Figure S1), and these four peaks were similar to the findings of previous studies [33].

**Figure 3:**
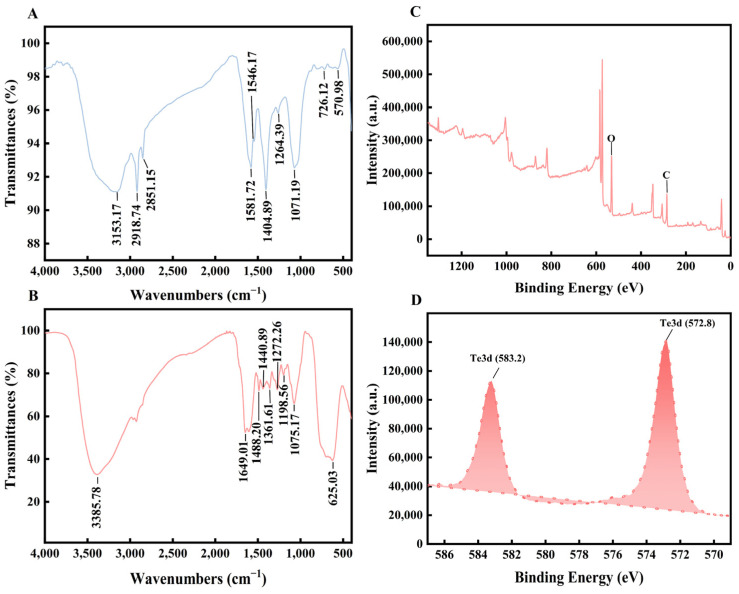
Nano characteristics of Bio-TeNPs: FTIR and XPS analyses. *M*. *oleifera* extract contained proteins, aromatic compounds, carboxylate groups, aliphatic acids, and inorganic ions (**A**). Bio-TeNPs contained proteins, aliphatic acids, and aromatic nitro compounds (**B**). Bio-TeNPs contained C, O, and Te(0) (**C**,**D**).

Based on the FTIR results, peaks were observed in the *M*. *oleifera* extract at 570.98 (C–I stretch), 726.12 (C-Cl stretch), 1071.19 (indicative of cyclic ethers or large rings containing carbon), 1264.39 (suggestive of aromatic primary amine, CN stretch), 1404.89 and 1564.17 (representing carboxylate groups), 1581.72 (related to secondary amine, NH bend), 2851.15 (associated with saturated aliphatic groups, methylene), and 3153.17 (indicative of ammonium ions) cm^−1^ (Figure 3A). Bio-TeNPs exhibited peaks at 625.03 (associated with alkyne, C–H bend), 1075.17 (related to the primary amine, C–N stretch), 1198.56 (indicative of tertiary amine, C–N stretch), 1272.26 (suggestive of hydroxyl groups, primary or secondary), 1361.61 (representing aliphatic nitro compounds), 1440.89 (associated with methyl groups, C–H stretch), 1488.20 (indicative of aromatic nitro compounds), 1649.01 (related to olefinic groups), and 3385.78 (associated with hydroxyl groups) cm^−1^ (Figure 3B). All the interpretations were based on an analysis of infrared spectra previously published in the literature [34]. These findings suggested that the *M*. *oleifera* extract contained proteins, aromatic compounds, carboxylate groups, aliphatic acids, and inorganic ions, while the Bio-TeNPs contained proteins, aliphatic acids, and aromatic nitro compounds. Notably, the proteins, aliphatic acids, and aromatic compounds present in the Bio-TeNPs originated from the *M*. *oleifera* extract. The XPS results also showed that the Bio-TeNPs contained many C and O elements (Figure 3C); this meant that the main components of the Bio-TeNPs were organic in nature. Furthermore, the presence of Te3d, associated with Te(0), was confirmed through XPS analysis (Figure 3D), and no Te(IV) peak was observed. This result indicated that Te(IV) was reduced to Te(0) by the *M. oleifera* extract and that Te(0) was an important constituent of Bio-TeNPs.

### 3.2. Antibacterial Activity of Bio-TeNPs

A zone of inhibition test was conducted to determine the antibacterial activity of the Bio-TeNPs. The results indicated that *M*. *oleifera* extract displayed noticeable antibacterial activity against *S*. *dysenteriae*, with slight effectiveness against *K*. *pneumoniae* and *E*. *coli* (Table 1). Chem-Te(0) demonstrated antibacterial efficacy against *S*. *dysenteriae*, *E*. *coli*, *K*. *pneumoniae*, *S*. *typhimurium*, *S*. *pneumoniae*, and *S*. *agalactiae* (Table 1). Bio-TeNPs exhibited excellent antibacterial activity against all tested pathogens except *S*. *aureus* (Table 1). Pictures of inhibition zones are shown in Figure S2. Though *M*. *oleifera* extract and Chem-Te(0) exhibited varied inhibitory effects against these bacteria, their levels of potency were generally lower than those of Bio-TeNPs at equivalent concentrations. This suggested that *M*. *oleifera* extract enhanced the antibacterial properties of Bio-TeNPs. Notably, *M*. *oleifera* extract by itself exhibited no antibacterial activity against *S*. *typhimurium*, *S*. *pneumonia*, and *S*. *agalactiae*, but it did enhance the antibacterial activity of Bio-TeNPs against these bacteria. Three explanations may be offered for these results, as follows: (1) It is possible that, during the synthesis of Bio-TeNPs, the active ingredients were enriched on the nanoparticles, resulting in higher concentrations on the Bio-TeNPs. *M*. *oleifera* extract has been reported to exhibit antibacterial ability against *S*. *typhimurium* [35]. However, no antibacterial effect against *S*. *typhimurium* was observed in our study. This might have been due to the active concentration of the extract. (2) The Bio-TeNPs were obtained using a typical production process involving biosynthesis methods and natural biological components [36]. These materials generally exhibit high biocompatibility which enhances the stability of the final product, increasing its persistence in biological systems, and improving its interaction with biological environments, thereby boosting its effectiveness. (3) The nanostructure of Bio-TeNPs might have facilitated easier penetration of bacteria, with its effectiveness being further enhanced by its multifaceted antibacterial mechanism [37].

### 3.3. Bio-TeNPs Inhibit Bacterial Growth

**Figure 4:**
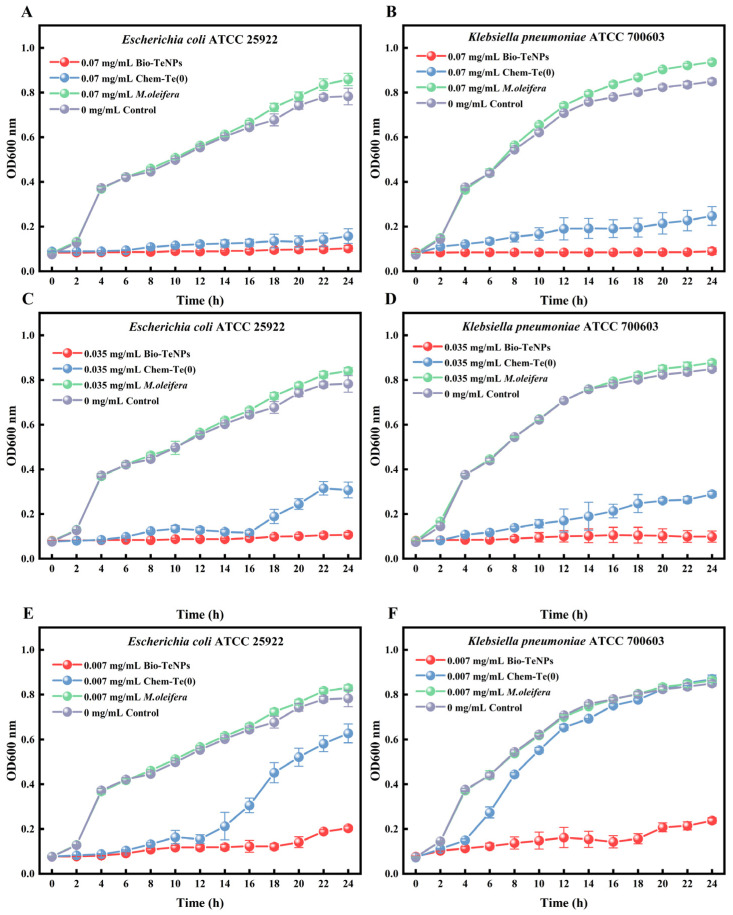
Effects of *M*. *oleifera* extract, Chem-Te(0), and Bio-TeNPs on the growth of *E*. *coli* (**A**,**C**,**E**) and *K*. *pneumoniae* (**B**,**D**,**F**) in MHB. Data are shown in the form of mean ± SD for three biological replicates.

To gain more insight into the antibacterial activity of Bio-TeNPs, *E. coli*, and *K*. *pneumoniae* were chosen for further evaluation via growth curve experiments. Bio-TeNPs at concentrations of 0.07, 0.035, and 0.007 mg/mL were selected for experimental purposes on account of their excellent antibacterial activity. The absorbance values of *E. coli* and *K*. *pneumoniae* increased with elapsed time in the control and *M*. *oleifera* extract group, while Bio-TeNPs and Chem-Te(0) showed different inhibitory effects on their growth (Figure 4). The results showed that Bio-TeNPs and Chem-Te(0) at 0.07 mg/mL were effective in rapidly preventing the proliferation of *E. coli*. At a concentration of 0.035 mg/mL, *E. coli* began to grow at 18 h with Chem-Te(0), whereas it remained entirely inhibited by Bio-TeNPs. When exposed to 0.007 mg/mL Chem-Te(0), *E. coli* growth was resumed at 16 h, while it was still predominantly inhibited at 0.07 mg/mL Bio-TeNPs (Figure 4A,C,E). Similarly, Bio-TeNPs (at 0.07 and 0.035 mg/mL) immediately inhibited the growth of *K*. *pneumoniae*, while Chem-Te(0) at the same concentration had a limited inhibitory effect on *K*. *pneumoniae*. Moreover, *K. pneumoniae* began to grow freely with Chem-Te(0) treatment at 4 h, while it was still inhibited by Bio-TeNPs at a concentration of 0.007 mg/mL (Figure 4B,D,F).

### 3.4. LSCM Observation of Bacteria Treated with Bio-TeNPs

**Figure 5:**
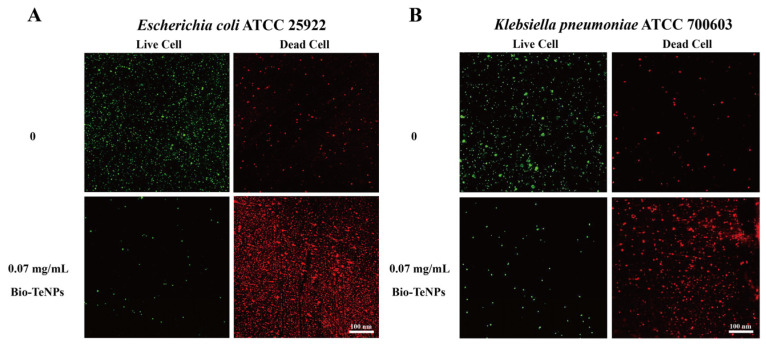
Live/dead cell staining of *E. coli* (**A**) and *K. pneumoniae* (**B**) cells with or without Bio-TeNPs treatment.

The antibacterial abilities of the Bio-TeNPs against *E. coli* and *K*. *pneumoniae* were further confirmed by microscopic observation. The percentage of dead cells was considerably higher, and that of live cells substantially lower, for *E. coli* and *K*. *pneumoniae* cells treated with 0.07 mg/mL of Bio-TeNPs, compared with corresponding values for untreated cells (Figure 5A,B). These findings indicated that the Bio-TeNPs exhibited excellent antibacterial ability.

### 3.5. Morphological Changes after Treatment with Bio-TeNPs

**Figure 6:**
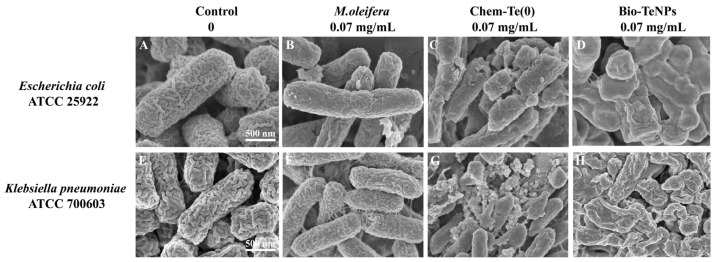
Morphological observation of *E. coli* and *K. pneumoniae*. SEM image of *E. coli* treated with ddH_2_0 (**A**), 0.07 mg/mL *M*. *oleifera* extract (**B**), 0.07 mg/mL Chem-Te(0) (**C**) and 0.07 mg/mL Bio-TeNPs (**D**). SEM image of *K. pneumoniae* treated with ddH_2_0 (**E**), 0.07 mg/mL *M*. *oleifera* extract (**F**), 0.07 mg/mL Chem-Te(0) (**G**) and 0.07 mg/mL Bio-TeNPs (**H**).

The morphologies of *E. coli* and *K. pneumoniae* cells under ddH_2_0, Chem-Te(0), and *M. oleifera* extract, and Bio-TeNPs treatments were observed via SEM. The results indicated that untreated *E. coli* and *K*. *pneumoniae* cells remained normal (Figure 6A,E). Cells treated with 0.07 mg/mL *M*. *oleifera* extract also showed no significant changes (Figure 6B,F). However, cells treated with 0.07 mg/mL Chem-Te(0) exhibited damage (Figure 6C,G), whereas the *E. coli* and *K*. *pneumoniae* cells treated with 0.07 mg/mL of Bio-TeNPs for 4 h were crumpled, broken, shrunken, and deformed (Figure 6D,H). The most significant cellular changes were observed after treatment with Bio-TeNPs. These results indicated that the *E. coli* and *K. pneumoniae* cells were destroyed by the Bio-TeNPs.

### 3.6. Anti-Biofilm Activity of Bio-TeNPs

**Figure 7:**
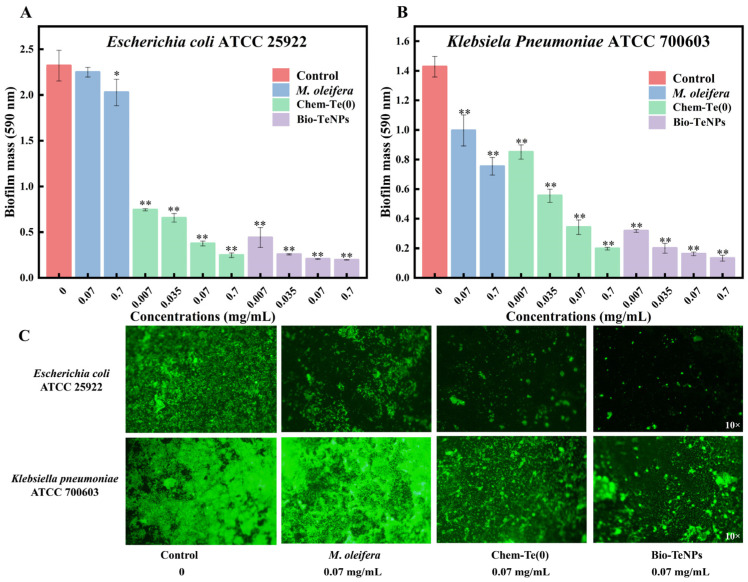
Effects of *M*. *oleifera* extract, Chem-Te(0), and Bio-TeNPs on biofilm formation of *E*. *coli* (**A**) and *K*. *pneumoniae* (**B**). The biofilm was observed using FITC staining at 0, 0.07 mg/mL *M*. *oleifera* extract, Chem-Te(0) and Bio-TeNPs (**C**). All data are presented in the form of mean ± standard deviation (SD) for three biological replicates. * indicates significance (*p* < 0.05); ** indicates high significance (*p* < 0.01).

Biofilm formation of *E*. *coli* and *K*. *pneumoniae* in 96-well plates was observed using the crystal violet staining method. The results revealed that *M*. *oleifera* extract exhibited significant anti-biofilm activity against *K*. *pneumoniae*, but no similar activity against *E*. *coli* was observed (Figure 7A,B). Both Chem-Te(0) and Bio-TeNPs demonstrated excellent antibacterial properties against *E*. *coli* and *K*. *pneumoniae*. With increased concentration, the inhibitory effect became more pronounced (Figure 7A,B), indicating the excellent anti-biofilm activity of Bio-TeNPs. At concentrations of 0.007 mg/mL and 0.035 mg/mL, Bio-TeNPs exhibited stronger anti-biofilm capabilities than Chem-Te(0). These results were further confirmed by the FITC staining observation. The complete *E*. *coli* and *K*. *pneumoniae* biofilms were observed at control, while biofilms were noticeably broken at 0.07 mg/mL Chem-Te(0) and Bio-TeNPs (Figure 7C).

### 3.7. Application of Bio-TeNPs against E. coli on the Surfaces of Glass Slides

**Figure 8:**
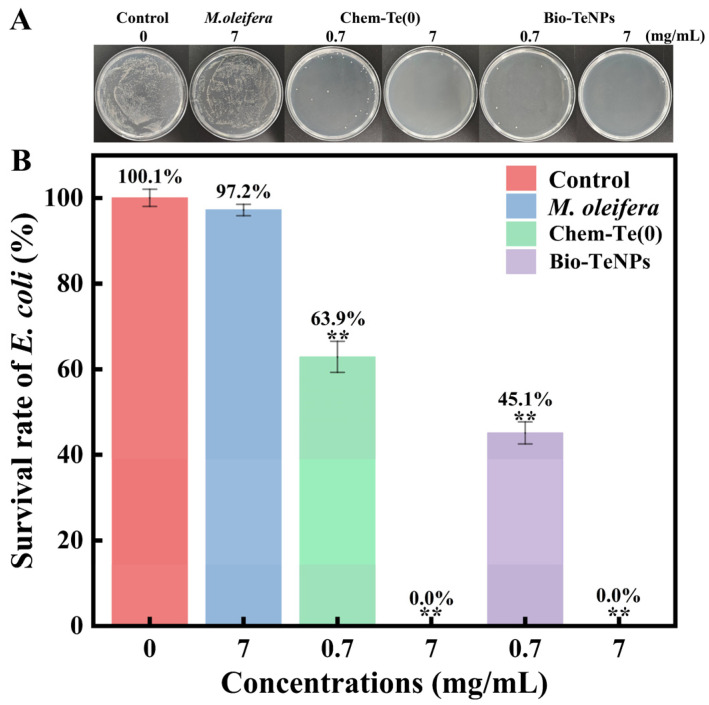
Clean efficiency of Bio-TeNPs against *E*. *coli* on the surface of glass slides. The coated plate (**A**) and survival rates (**B**) of *E*. *coli* cells treated with normal saline, *M*. *oleifera* extract, Chem-Te(0), and Bio-TeNPs. The results are expressed as the mean ± standard deviation (SD) of three biological replicates. Asterisks denote significance levels (** for *p* < 0.01).

*E. coli* often adsorbs to the surfaces of solid media in the environment, enabling it to survive, and resulting in contamination. In this study, *E. coli* was coated onto the surface of a glass slide to test the antibacterial application of Bio-TeNPs. The survival rates of *E. coli* cells treated with Bio-TeNPs and normal saline were calculated on the MHA plates (Figure 8A). It was found that the survival rates of *E. coli* after treatment with normal saline, 7 mg/mL *M*. *oleifera* extract, 0.7 and 7 mg/mL of Chem-Te(0), and 0.7 and 7 mg/mL of Bio-TeNPs were 100%, 97.2%, 63.9%, 0, 45.1%, and 0, respectively (Figure 8B). These findings indicated that 7 mg/mL Bio-TeNPs and Chem-Te(0) exhibited great potential for antibacterial application, while the cleaning ability of Bio-TeNPs was higher than that of Chem-Te(0) at a concentration of 0.7 mg/mL.

## 4. Discussion

In the present study, the color, morphology, and size of Bio-TeNPs were observed through the visible absorbance spectrum, DLS, TEM, and SEM. Bio-TeNPs exhibited a number of absorption peaks at 230 nm, 250 nm, 260 nm, and 350 nm and their color was typically black. Consistent with results obtained in prior related studies, the synthesized Te nanoparticles were also black [38,39]. However, the absorption peaks of the Te nanoparticles were different. Our Bio-TeNPs have multiple absorption peaks at 200–300 nm, probably due to the complex composition of *M*. *oleifera* extract. The Bio-TeNPs produced by *Shewanella baltica* showed an absorption peak at 210 nm [40], whereas those synthesized using tea extracts showed an absorption peak at 270 nm [41]. This difference in spectra may be due to the different constituents of Bio-TeNPs. In addition, the concentration of Bio-TeNPs might also cause fluctuations in absorption peaks. Previous studies have shown that absorption bands may be related to the size of Te nanoparticles [42]. The DLS, TEM, and SEM results revealed that the synthesized Bio-TeNPs exhibited nearly spherical shapes with diameters of approximately 20–50 nm. These shapes and sizes are similar to those of the AgNPs, AuNPs, ZnONPs, and FeONPs synthesized by *M*. *oleifera* in a previous study [43]. Previous research has also shown that differences in plant/microbial species and synthesis conditions can cause changes in the shape and size of Bio-TeNPs [24,44].

Regarding the constituents of Bio-TeNPs, FTIR analysis revealed that *M*. *oleifera* extract contained proteins, aromatic compounds, carboxylate groups, aliphatic acids, and inorganic ions, in line with previous reports [45]. Bio-TeNPs were found to contain proteins, aliphatic acids, and aromatic nitro compounds originating from *M*. *oleifera* extract. This finding is in line with the composition of AgNPs, AuNPs, ZnONPs, and FeONPs produced by *M*. *oleifera* [43]. However, there may be variations in the composition and content of these substances. Moreover, our XPS results indicated that Bio-TeNPs contained organic elements such as C, O, and Te(0). The presence of Te(0) suggests reduction by substances such as cysteine [16] in the *M*. *oleifera* extract from K_2_TeO_3_, while C and O were derived from organic compounds in the *M*. *oleifera* extract. Similarly, Te(0) has been identified as a primary constituent in Bio-TeNPs produced by microorganisms and other plants [26,31,46].

The antibacterial spectrum and efficacy of Bio-TeNPs recorded in the present work surpassed that reported in some previous studies. Regarding the antibacterial spectrum, Bio-TeNPs produced by *Aspergillus welwitschiae* have previously been shown to inhibit *E*. *coli* (with an inhibition zone of 29 mm at 25 mg/mL) but not *Klebsiella* [38]. However, in this study, Bio-TeNPs exhibited antibacterial activity against *E*. *coli*, *K*. *pneumoniae*, *S*. *dysenteriae*, *S*. *typhimurium*, *S*. *pneumoniae*, and *S*. *agalactiae*. Concerning antibacterial efficacy, Bio-TeNPs synthesized by *Mortierella* sp. AB1 was shown to inhibit *E*. *coli* (with an inhibition zone of 32.3 ± 0.9 mm at 10 mg/mL), *S*. *dysenteriae* (with an inhibition zone of 32.3 ± 1.2 mm at 10 mg/mL), and *S*. *typhimurium* (with an inhibition zone of 29.7 ± 1.5 mm at 10 mg/mL) [26]. Nonetheless, the antibacterial potency of Bio-TeNPs in this investigation surpassed that of both *A*. *welwitschiae* and *Mortierella* sp. AB1. This might be due to the antibacterial constituents of *M. oleifera*, which is an edible and medicinal plant [47]. The antibacterial activity of Bio-TeNPs recorded in the present study is comparable to that of AgNPs produced by *Gracilaria crassa* [48] and better than AgNPs produced by citrus sinensis peel extract [49]. Compared with *M. oleifera*-based nanomaterials, Bio-TeNPs in the present study showed antibacterial activity which was higher than that of AgNPs [50] and ZnONPs [51], but lower than that of iron oxide [52] nanoparticles. In the present study, Bio-TeNPs were also found to disrupt the integrity of *E*. *coli* and *K*. *pneumoniae* membranes and alter bacterial morphology. Interestingly, the positively charged Bio-TeNPs might be an important cause of the above phenomenon. Previous studies have indicated that one of the antibacterial mechanisms of biomaterials is the adsorption of positively charged nanoparticles onto negatively charged bacterial surfaces through electrostatic interaction, resulting in membrane damage and bacterial morphological changes [53]. Bar et al. also found that the interaction between different quantum dot materials and SLBs was driven by electrostatic attraction, and that defective SLBs would adsorb quantum dots more easily [54]. On the other hand, oxidative stress is also an important antibacterial mechanism, and positively charged selenium nanoparticles [55] and iron oxide nanoparticles [52] could kill bacteria through oxidative stress, contact with nanomaterials causing peroxidation of membrane phospholipids thus directly destroying the integrity of the cell membrane [53]. Moreover, Bio-TeNPs in this study showed noticeable anti-biofilm activity against *E*. *coli* and *K*. *pneumoniae*. This has rarely been reported for other Bio-TeNPs; indeed, at the time of writing, no Bio-TeNPs have been reported to exhibit anti-biofilm activity. However, extracts of pepper [30], aloe vera [29], and citric juice [31] have been reported to synthesize Bio-TeNPs against MDR *E. coli* and MRSA. Interestingly, the Bio-TeNPs in the present study exhibited no antibacterial activity against *S. aureus*; in addition, the antibacterial effect against Gram-negative bacteria was stronger than that against Gram-positive bacteria. This suggests that the antibacterial mechanism of biogenic tellurium nanoparticles may be somewhat unique, and different from that of Bio-TeNPs produced by other plants. Additionally, the toxicity of Bio-TeNPs was evaluated on Vero-E6 cells. No cytotoxicity was observed at concentrations of 0.7 and 0.07 mg/mL and there was a certain level of toxicity at a concentration of 7 mg/mL (Figure S3). In the present study, aqueous extract obtained from thin branches was used. The branches of *M*. *oleifera* are typically discarded after maturity, as they have lower economic value compared to the seeds and flowers. The use of such material may help save on the cost of synthesis in the future.

## 5. Conclusions

In this study, *M*. *oleifera* extract was used to synthesize Bio-TeNPs. The produced Bio-TeNPs consisted of Te(0) and some organics from *M*. *oleifera* with the diameter ranging from 20 to 50 nm. The Bio-TeNPs showed excellent antibacterial activity against various pathogens based on the results of inhibition zone tests. Further, the inhibitory capacity of Bio-TeNPs against *E. coli* and *K. pneumoniae* was demonstrated by the growth curve experiments, live/dead cell staining, and cell morphology observation. The biofilm formation of *E*. *coli* and *K*. *pneumoniae* was also noticeably inhibited by Bio-TeNPs. Additionally, the Bio-TeNPs demonstrated significant antibacterial efficacy against *E*. *coli* on glass slides, highlighting their potential for antibacterial applications. Bio-TeNPs have been proven to be highly effective antibacterial materials, suitable for various medical and environmental sanitation applications based on the results in this study. However, the antibacterial mechanism of Bio-TeNPs remains unknown and requires further investigation. Additionally, the obtained Bio-TeNPs tend to aggregate easily. In the future, the addition of dispersants and stabilizers may be considered to enhance their stability and homogeneity.

## 6. Patents

Chinese patent application number: ZL 2022 1 0216514.5.

## Figures and Tables

**Table 1:** Antibacterial activity of Bio-TeNPs on bacterial pathogens.

Strains	G^+^/G^−^	*M. oleifera*	Chem-Te(0)	Bio-TeNPs		
		7 mg/mL	7 mg/mL	7 mg/mL	0.7 mg/mL	0.07 mg/mL
*Shigella dysenteriae* CMCC 51252	G^−^	19.0 ± 1.0	42.5 ± 2.5	53.3 ± 0.5	46.0 ± 0.8	44.0 ± 0.8
*Salmonella typhimurium* ATCC 14028	G^−^	ND	32.0 ± 0.8	43.3 ± 1.3	41.3 ± 1.9	38.0 ± 2.2
*Escherichia coli* ATCC 25922	G^−^	ND	30.0 ± 1.6	48.7 ± 1.9	45.3 ± 0.5	42.0 ± 1.6
*Klebsiella pneumoniae* ATCC 700603	G^−^	9.3 ± 1.9	27.0 ± 1.4	43.0 ± 2.1	40.3 ± 0.5	37.7 ± 0.9
*Streptococcus pneumoniae* ATCC 49619	G^+^	ND	15.3 ± 0.5	29.7 ± 3.1	28.7 ± 3.4	26.7 ± 0.5
*Streptococcus agalactiae* E442	G^+^	ND	11.0 ± 0.8	26.0 ± 2.2	22.0 ± 0.8	18.7 ± 0.9
*Staphylococcus aureus* ATCC 25923	G^+^	ND	ND	ND	ND	ND

Units are mm; the outer diameter of the Oxford cup is 8 mm; ND stands for “no inhibition zone detected”; data are shown in the form of mean ± SD (*n* = 3).

## Data Availability

The original contributions presented in the study are included in the article/Supplementary Material, further inquiries can be directed to the corresponding authors.

## Supplementary Materials

The following supporting information can be downloaded at: https://www.mdpi.com/article/10.3390/microorganisms12091847/s1, Figure S1. The XRD of Bio-TeNPs. Figure S2. Inhibition zone pictures of antibacterial activity of Bio-TeNPs against bacterial pathogens. Figure S3. The cytotoxicity of Bio-TeNPs.
