# Supplementary material for: Synthesis of Tellurium Nanoparticles Using Moringa oleifera Extract, and Their Antibacterial and Antibiofilm Effects against Bacterial Pathogens
Source: Microorganisms. 2024 Sep 6;12(9):1847. doi: 10.3390/microorganisms12091847 (PMC11434551; doi:10.3390/microorganisms12091847)
Supplement: Supplementary file 1 [file microorganisms-12-01847-s001.zip › microorganisms-3102489-supplementary.pdf]

Supporting Information

Synthesis of Tellurium Nanoparticles Using *Moringa oleifera* Extract, and their Antibacterial and Antibiofilm Effects against Bacterial Pathogens

Bo Ao [1,2,†], Honglin Jiang [3,†], Xuan Cai [4], Decheng Liu [1], Junming Tu [1], Xiaoshan Shi [1], Yanxiang Wang [1], Fei He [3], Jing Lv [3], Jingjing Li [1], Yuanliang Hu [1], Xian Xia [1,2,*] and Jianjun Hou [1,*]

[1] Hubei Key Laboratory of Edible Wild Plants Conservation & Utilization, Hubei Engineering Research Center of Characteristic Wild Vegetable Breeding and Comprehensive Utilization Technology, Hubei Normal University, Huangshi 435002, China; aobo99123@163.com (B.A.); 15667172820@163.com (D.L.); junming_tu@hbnu.edu.cn (J.T.); shixs@hbnu.edu.cn (X.S.); wangyx@hbnu.edu.cn (Y.W.); pandali1980@hotmail.com (J.L.); ylhu@hbnu.edu.cn (Y.H.)

[2] Hubei Key Laboratory of Natural Medicinal Chemistry and Resource Evaluation, School of Pharmacy, Tongji Medical College, Huazhong University of Science and Technology, Wuhan 430030, China

[3] Hubei Provincial Center for Disease Control and Prevention, Wuhan 430079, China; jhl13098825387@163.com (H.J.); 13036167687@163.com (F.H.); lvjing979899@163.com (J.L.)

[4] Wuhan University, Wuhan 430060, China; rm001319@whu.edu.cn

* Correspondence: xianxia@hbnu.edu.cn (X.X.); jjhou@hbnu.edu.cn (J.H.)

† These authors contributed equally to this work.

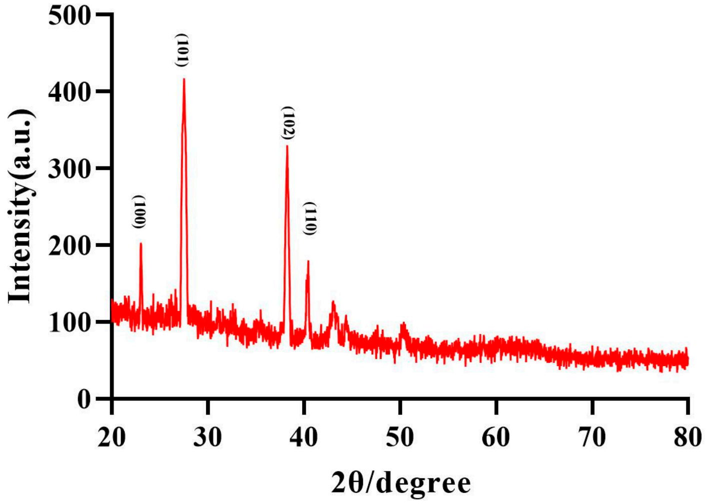

Figure S1. The XRD of Bio-TeNPs.

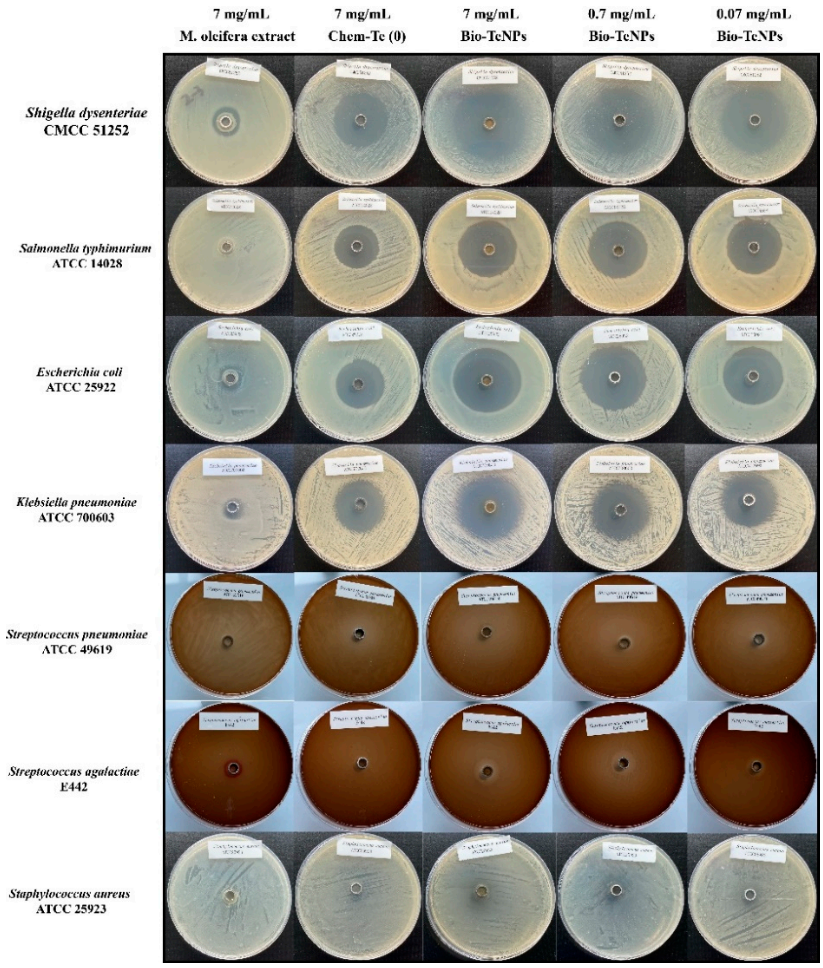

Figure S2. Inhibition zone pictures of antibacterial activity of Bio-TeNPs against bacterial pathogens.

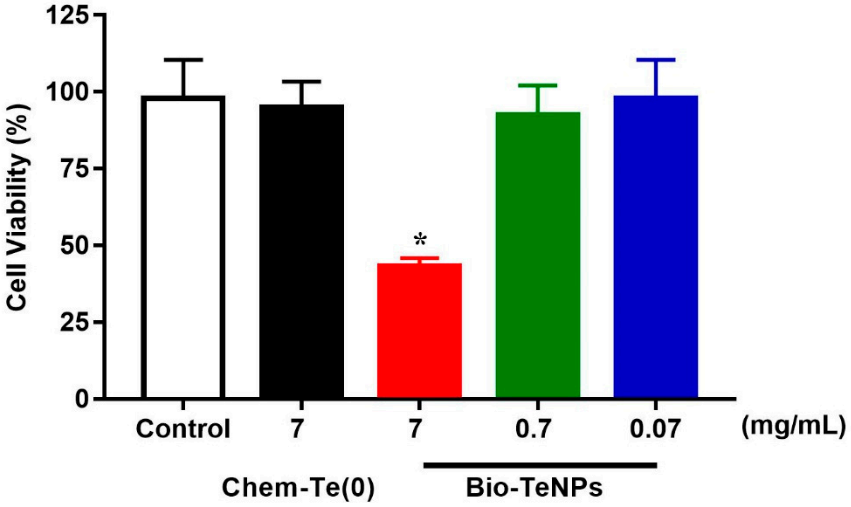

Figure S3. The cytotoxicity of Bio-TeNPs.
